# Digital solutions, real-world challenges: lessons from mHealth trials in oncology

**DOI:** 10.3389/fdgth.2025.1721363

**Published:** 2026-02-05

**Authors:** Dominique G. Stuijt, Igor Radanovic, Vasileios Exadaktylos, Ellen Kapiteijn, Tom van der Hulle, Jorg R. Oddens, Erik van Gennep, Lois A. Daamen, Marieke A. R. Bak, M. Corrette Ploem, Martijn G. H. van Oijen, Adriaan D. Bins, Jacobus J. Bosch

**Affiliations:** 1 Department of Medical Oncology, Amsterdam University Medical Centers, University of Amsterdam, Amsterdam, Netherlands; 2 Centre for Human Drug Research, Leiden, Netherlands; 3 Cancer Center Amsterdam, Theme Therapy, Amsterdam, Netherlands; 4 Department of Medical Oncology, Leiden University Medical Center, Leiden, Netherlands; 5 Department of Urology, Amsterdam University Medical Centers, University of Amsterdam, Amsterdam, Netherlands; 6 Department of Urology, Leiden University Medical Center, Leiden, Netherlands; 7 Division of Imaging & Oncology, University Medical Center Utrecht, Utrecht, Netherlands; 8 Department of Ethics, Law and Humanities, Amsterdam UMC, University of Amsterdam, Amsterdam, Netherlands

**Keywords:** challenges, mHealth, oncology, remote monitoring, smartphone, wearable

## Abstract

The use of mobile health (mHealth) technologies in oncology, such as wearable devices and smartphone applications, is gaining momentum due to their potential to improve quality of life, enhance treatment adherence, and positively impact survival outcomes for cancer patients. However, as a relatively new and evolving field, mHealth research faces a set of challenges in both study design and implementation. This article identifies key obstacles by drawing on preliminary experience from three mHealth studies in oncology: the eBladder study, the CHOPIN study, and the LAPSTAR study (ongoing studies at publication date). The topics covered are clustered into four categories: (1) planning and design (e.g., determining appropriate follow-up durations and inclusion criteria, defining digital support as an endpoint, developing response windows for digital questionnaires, establishing active measurement frequency); (2) technology set-up and study execution (e.g., aligning treatment and mHealth schedules, managing treatment heterogeneity and changes, establishing device configuration, scheduling data checks, determining end-of-study visits); (3) adherence (e.g., developing integrated platforms, balancing passive and active measurements, considering treatment goals as motivators, evaluating mHealth literacy); and (4) data reliability (capturing adverse events in real-time, ensuring device accuracy, and privacy considerations). This article also contains some practical recommendations in response to these challenges, meant to inspire researchers who are embarking on future mHealth studies in oncology.

**Clinical Trial Registration:**

https://onderzoekmetmensen.nl/en, identifiers NL81928.029.22 (eBladder trial), NL69508.058.19 (CHOPIN trial), and NL85622.041.24 (LAPSTAR trial).

## 1 Introduction

**Figure 1:**
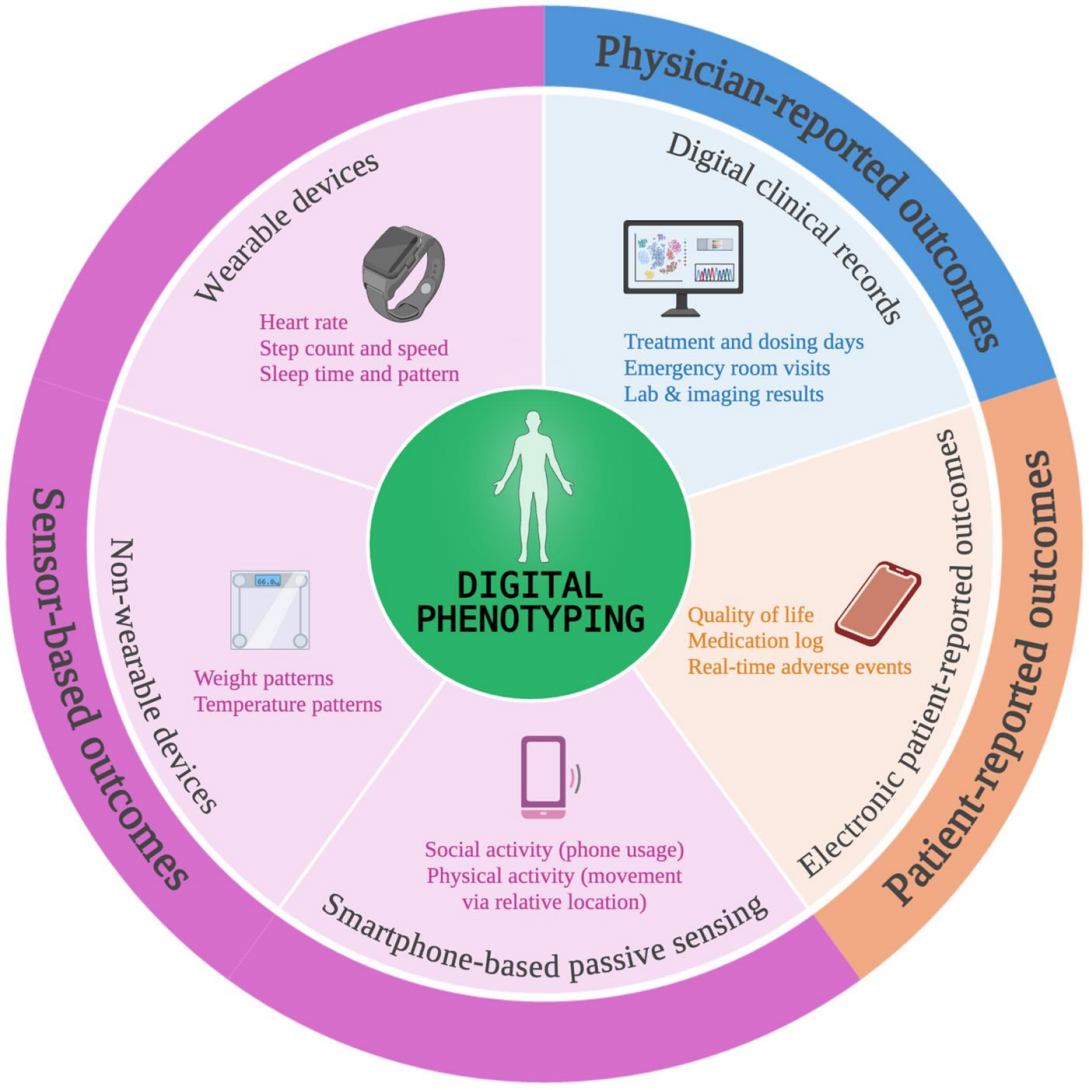
mHealth devices and digital phenotyping. This figure illustrates various data inputs that contribute to the digital phenotyping of a patient. These include mHealth technologies such as wearable and non-wearable devices, smartphone-based passive sensing, and electronic patient-reported outcome platforms. Additionally, clinical data from digital hospital records remains essential for the translation of the digital endpoints. Note that these are illustrative examples and should not be interpreted as a complete enumeration of all digital endpoints. Created in https://BioRender.com.

Mobile health (mHealth) refers to the use of mobile devices and sensors to facilitate continuous and ubiquitous healthcare (1). It can include smartphones or tablets with their respective apps, wearable devices such as smartwatches, and non-wearable smart devices such as scales or thermometers (Figure 1). mHealth has proven to be a useful tool for improving cancer patients' quality of life, therapeutic adherence, and survival outcomes (2–5). Usage of these devices is already widespread, as is the willingness of cancer patients to adopt them (6, 7).

This high level of engagement creates an opportunity for further research into the potential benefits of mHealth in healthcare. Additionally, the integration of machine learning with mHealth data adds value, as already hinted by its ability to predict hospitalizations using simple data inputs such as step count (8, 9). This finding, achieved with a single input, alludes to the vast potential of integrated mHealth platforms which include multiple variables, such as electronic patient-reported outcomes (ePRO), wearable sensor data, and smartphone-based passive sensing (10, 11). Pooling of such data could result in digital phenotyping of the patient, which refers to the process of using data from digital devices to measure and analyze behavioral and physiological patterns in real time (12). This can provide insights into a patients' mental and physical health (13, 14). Digital phenotyping of patients with cancer promises to unlock new and more personalized treatments. An example is the emerging concept of *digital twins*, in which multimodal data is integrated from different sources like electronic health records, imaging, genomic and sensor data, into a virtual replica or model to run simulations and predict outcomes (15, 16). This could mean enhanced precision oncology and personalized care, though the complete range of its applications remains to be seen.

Additionally, mHealth ideally provides a seamless continuum across medical disciplines, facilitating comprehensive clinical pathways for patients. For instance, bladder cancer patients may begin their treatment with the urological team during the diagnostic phase but eventually need to be transferred to medical oncology for systemic treatment. Or vice versa, starting with neoadjuvant therapy and ultimately transitioning to urological care for a radical cystectomy. In these cases, mHealth supports an integrative approach through their whole anti-cancer treatment journey. This allows different specialists to follow physiological patterns such as fitness and weight loss in given periods of time, improving decision-making.

To fully realize mHealth's potential, further research is essential to ensure reliability and clinical validation and to prove efficacy (17). However, as a relatively new field, researchers face numerous challenges in designing and executing mHealth studies (18–22), which can lead to issues such as missing data, reduced study reliability, and even concerns over data security and patient privacy. In a survey to authors of mHealth studies, the most reported methodological challenges were ensuring the intervention was implemented as intended and defining and measuring adherence (17). Similarly, in a review that summarizes the technical solutions and credibility of digital twins in oncology, the challenges mentioned are substantial resources, digitization of data, appropriate validation and ensuring end-user acceptance (16). While these challenges are likely valid and worth considering, they remain broad and do not address the practical, day-to-day difficulties researchers encounter. Similarly, they overlook the specific issues related to patients and treatments in oncology. To our knowledge, no studies have yet been published focusing on the operational challenges of conducting mHealth studies specifically within the oncology field. In this article, we aim to identify and examine both the challenges we have faced in our own research and those reported in the literature. Our goal is to support future researchers in designing more robust, consistent, and reliable study protocols, ultimately advancing the quality of mHealth research in oncology.

## 2 Evaluated mHealth studies

Three oncology studies using mHealth digital endpoint collection were evaluated: eBladder (bladder cancer), CHOPIN (uveal melanoma), and LAPSTAR (pancreatic duct carcinoma). These studies serve as illustrative examples for those less familiar with mHealth trial designs, helping to highlight the challenges encountered in their conduct.

### 2.1 General aspects

The eBladder study is an observational study to explore the feasibility of an mHealth platform in bladder cancer patients during their standard-of-care cancer treatment with curative intent.

The CHOPIN study is an interventional phase 2 study to test the safety, feasibility and efficacy of combining percutaneous hepatic perfusion with ipilimumab and nivolumab in uveal melanoma patients with liver metastasis, with optional participation of remote monitoring with an mHealth platform to assess treatment impact and quality of life (23).

The LAPSTAR study is a randomized controlled study to evaluate local ablative treatment with magnetic resonance-guided radiotherapy in pancreatic ductal adenocarcinoma patients who are not eligible for tumor resection. Participation in remote monitoring with an mHealth platform to assess treatment impact and quality of life is optional.

All three studies involved academic medical centers from the Netherlands which concentrate complex oncology care and clinical trials. See Table 1 for further administrative information of the three studies, including full title, trial registration, funding, and sponsor information.

**Table 1:** Administrative information and summary of the three mHealth oncology studies discussed in this article.

Short title		eBladder	CHOPIN	LAPSTAR
Administrative information				
Full title		An observational, non-interventional cohort study to monitor physical and social activity of bladder cancer patients during treatment with curative intent by using conventional and digital methodology	Phase 2 study combining hepatic percutaneous perfusion with ipilimumab plus nivolumab in advanced uveal melanoma	Locally advanced pancreatic cancer after systemic therapy: ablative MR-guided radiotherapy
Status		Actively recruiting and enrolling patients	All subjects enrolled, data collection still ongoing	Actively recruiting and enrolling patients
Trial registry	EU-CTR (56)	–	CTIS2024-516127-14-01 CTIS2024-516125-31-02	–
	https://www.ClinicalTrial.gov (57)	–	NCT04283890	NCT06272162
	CCMO (58)	NL81928.029.22	NL69508.058.19	NL85622.041.24
	ISRCTN (59)	ISRCTN55744047	–	–
Trial sponsor		Academisch Medisch Centrum (investigator-initiated study)	Leiden University Medical Center (investigator-initiated study)	University Medical Center Utrecht (investigator-initiated study)
Trial sites		2 academic hospitals in the Netherlands	1 academic hospital in the Netherlands	15 academic and non-academic hospitals in the Netherlands
Funding		In kind contribution from the KWF Kankerbestrijding grant number 13144 and the Centre for Human Drug Research foundation	In kind contribution from Bristol-Myerss Squibb and Delcath Systems Inc.	KWF Kankerbestrijding grant number 15030
Study design				
Study type		Observational (mHealth monitoring during standard-of-care treatment)	Interventional (clinical study with optional mHealth digital monitoring)	Interventional (clinical study with optional mHealth digital monitoring)
Study population		Patients with muscle and non-muscle invasive bladder cancer	Patients with unresectable hepatic metastases of uveal melanoma with or without limited extrahepatic disease	Patients with locally advanced pancreatic cancer who are not eligible for tumor resection after chemotherapy
Population size		44 patients included (goal 45 patients)	19 of the 28 trial participants opted for remote monitoring	18 of the 33 trial participants opted for remote monitoring^a^
Demographics		39 men, 5 women Mean age 67 years, range 48–86	12 men, 7 women Mean age 63, range 39–75	4 men, 15 women Mean age 67, 52–80
Treatment's objective		Curative	Palliative	Palliative
mHealth-related objectives		Determine the feasibility of monitoring bladder cancer patients with the Trial@home platform Correlate social and physical data with quality-of-life and performance status Exploratory: determine effects of cancer therapy, compare different treatment groups, determine association between digital data and adverse events or hospital admissions	Assess the feasibility of using the digital platform within an interventional trial Assess quality of life and physical activity Exploratively generate new digital biomarkers	Assess the feasibility of using the digital platform within an interventional trial Assess quality of life and physical activity Exploratively generate new digital biomarkers
Data collection duration		12–32 weeks	6 months	18 months
mHealth tools used (Trial@home platform)^b^		Smartwatch Smart scale Smart thermometer Smart sleep sensor CHDR MORE app ePRO app	Smartwatch Smart scale ePRO app	Smartwatch Smart scale Smart sleep sensor CHDR MORE app ePRO app

a This trial was actively recruiting and still including patients at the moment of the writing of this article.

b The choice of mHealth tool was based on factors such as the study population, cancer type, study objectives, and budget. As a result, the three studies did not all use the same devices.

### 2.2 mHealth platform

All three studies used the Trial@home platform as mHealth tool to collect the digital endpoints. Trial@home comprises of wearable and non-wearable devices, and smartphone apps. More specifically, the devices by Withings®, namely a smartwatch (Steel HR), a scale (Body+), a thermometer (Thermo), and a sleep sensor (Sleep Analyzer). These devices are connected via the “Withings Healthmate” app to the patients' smartphone for data collection and visualization.

The Trial@home platform also includes two other apps: the “CHDR MORE” app, a smartphone-based passive sensing app which collects phone and app usage data from the patients' phone, and the “Promasys ePRO” app, which is used for electronic patient-reported outcomes. This app allows administering the appropriate questionnaire and its frequency according to the study objectives and protocol.

Table 1 shows which of these devices and apps are used per trial. For these studies, not having a (compatible) smartphone was not an exclusion criterion, since in those cases, the study team would lend one to the participant. However, then the social activity via the “CHDR MORE” app would not be collected since there would be no representative smartphone use (i.e., app usage such as social media apps).

The digital endpoints collected per study are displayed in Table 2. At the time of writing, the eBladder and LAPSTAR are still ongoing, while the CHOPIN study has recently finished its inclusion, though its results are not yet published.

**Table 2:** Digital endpoints collected in the three oncology mHealth trials.

Source/device	Parameter collected	Frequency of data collection	Included in trials^a^
Physician-reported outcomes			
Medical file	Treatment type and dates	Collected if present	E, C, L
	Number of treatment interruptions and stop of treatment (and reason)		E, C, L
	Number of emergency room visits (and reason)		E
	Number of planned and unplanned hospitalizations (and reason, date, length)		E
	Adverse (device) events		E, L
	Radiological evaluations (and date)		E, L
Measured by investigator	Performance status according to the Eastern Cooperative Oncology Group (ECOG) (35)	Baseline and end-of-study	E, C, L
mHealth platform sensor outcomes (Trial@home platform)			
Withings Steel HR smartwatch	Step count (steps per minute) Heart rate (beats per minute) Sleep time (hours) Compliance rates (wear-time: hours per day)	Continuously	E, C, L
Withings Body + Scale	Weight (kg) Body composition (%) Compliance rates	Weekly	E, C, L
Withings Thermo	Temperature (°C) Compliance rates	Daily	E
Withings Sleep	Sleep time and pattern (sleep time, time in bed, sleep phases) Compliance rates	Continuously	E, L
MORE app (smartphone)	Social activity: ⚬ Phone usage (length of call, last 3 digits of phone number, number known/unknown) ⚬ App usage (categories of apps, start time, running in background/ foreground) Physical activity: relative location (GPS)	Continuously	E, L
Electronic patient-reported outcomes			
ePRO app (smartphone)	Quality of Life of Cancer Patients’ questionnaire [e.g., EORTC QLQ-C30 (35)]	Weekly (E), less frequently (C, L)	E, C, L
	International Prostate Symptom Score (IPSS) (60)	Every 4 weeks	E
	Urinary frequency log	Daily	E
	Pain medication log	If applicable	L
	Experience and subjective burden questionnaire	End of study	E

a E, eBladder study; C, CHOPIN study; L, LAPSTAR study.

### 2.3 Operational details

**Figure 2:**
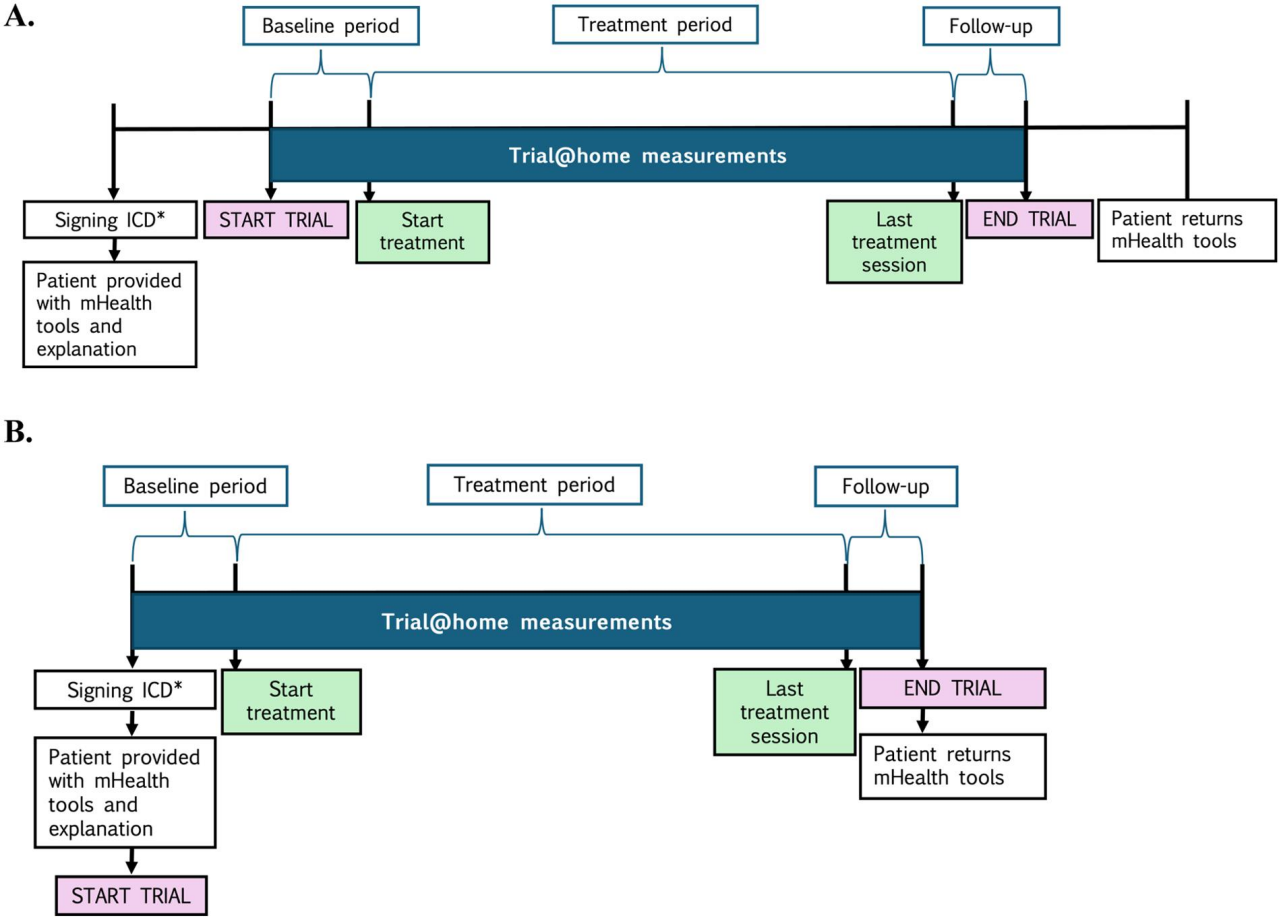
General mHealth study design of the eBladder, CHOPIN and LAPSTAR studies. *ICD, informed consent document. This figure illustrates the study design implemented across the three mHealth studies. Two approaches were used. In figure **(A)**, participants signed the ICD and then waited for the predetermined baseline period (i.e., two weeks prior to the first treatment day). While this method was intended to avoid baseline duration differences, logistical constraints sometimes led to short-notice scheduling, resulting in shorter actual baselines. In figure **(B)**, baseline measurements commenced on the same day the ICD was signed. Although this can result in longer and more variable baseline durations, we consider this approach preferable due to its practicality and potential for richer baseline data. The CHOPIN and LAPSTAR study used approach **(A)**, since the mHealth researcher was not present during the signing of the ICD. The eBladder study used approach **(B)**.

After obtaining written informed consent, patients are provided with their mHealth devices and respective apps (see sub-section “*mHealth platform*”), according to the study protocol. Subsequently, an explanation is given about the use of the devices and apps. The research team is available to provide technical assistance for the duration of the study. The measurement of digital endpoints from the study devices starts with the allocation of study devices or as stipulated by the study protocol (see Figure 2 for the general study timeline). Patients are instructed to wear the smartwatch as much as possible during waking hours and during sleep, to measure their weight and temperature according to the frequency stipulated per study protocol (see Table 2) and to fill in their questionnaires via the ePRO app. After inclusion, the patient continues to use the mHealth devices until the end of the study or until they withdraw consent. At the end of the study, the patients are asked to hand in the devices, and to fill in an experience and subjective burden questionnaire to evaluate their experiences with the devices and apps.

## 3 Operational challenges of mHealth studies in oncology

In this section we discuss the operational challenges encountered in the eBladder, CHOPIN, and LAPSTAR studies. The challenges were collected mainly through the first author, who worked directly with patients in all three studies. The remaining authors, each involved in one or more of the studies, checked, confirmed, or added their own experiences. We included only those challenges that arose repeatedly and across multiple studies to avoid emphasizing study-specific issues and to highlight patterns that may be generalizable and informative for other mHealth studies.

The challenges are grouped into four categories: (1) those arising during study set-up and design, (2) those encountered during technology implementation and study execution, (3) challenges related to adherence, and (4) data reliability and privacy considerations.

### 3.1 Challenges during study set-up and design

#### 3.1.1 Determining appropriate follow-up periods

Although there has been a recent increase in the number of mHealth studies (24), much remains unknown in the field of digital health data. As a result, making informed study design decisions, such as determining appropriate follow-up periods, is often challenging due to the limited evidence available (1, 25). For example, in the eBladder study, for comparing the effects of systemic therapy on daily physical activity vs. surgical treatment, a standard 4-week follow-up was chosen since recovery trajectories in bladder cancer patients are not well described in the literature. However, we have observed that some digital parameters, such as steps or heart rate, do not always return to baseline within the established follow-up period. This raises important questions: Do these parameters ever return to baseline? If so, how long does that process take? To be able to deal with this situation, we recommend reviewing existing literature to check for similar studies. In the absence of clear evidence, extending the study duration some weeks from the expected clinical recovery could be a reasonable approach, given that participants often report low burden in long-term monitoring (6).

#### 3.1.2 In- and exclusion criteria: gait impairment

The inclusion of patients with walking impairments (e.g., use of crutches, rollator or wheelchair) to mHealth studies, where main endpoints include step counts and fitness levels, are a point of debate (26). Excluding these patients introduces bias, but their data can also skew results due to being outliers. For instance, patients in wheelchairs will show no change in steps over time, offering no meaningful data on this parameter. However, patients with partial gait impairment, such as those with chemotherapy-induced peripheral neuropathy, may still provide useful insights into walking patterns. However, given the large sample size required to accurately account for such cases in the machine learning models (27), and the current low recruitment rates (28), we recommend excluding these patients in the early stages of studies. To avoid the ethical implications of systematically excluding less mobile patients and thereby limiting mHealth applicability to only the fittest individuals, subsequent research phases, ideally with larger cohorts and potentially tailored software, should specifically include gait-impaired populations. It is also important to consider the possibility of initially fit participants becoming wheelchair-bound after treatment. When analyzing the dataset which include such cases, we suggest that subjects should not be immediately excluded as outliers, but clinical data must be used to correct for these changes.

We emphasize the gait criteria given the importance of step count and its derivatives as mHealth-related endpoints. Nonetheless, several other potential exclusion criteria could also be considered, such as visual impairments that may affect interaction with the apps or tremors that could interfere with movement measurements. These are all important and interesting aspects to explore. However, we do not address them here because we did not encounter these issues in our studies.

#### 3.1.3 Contact for digital support as study endpoint

In most of mHealth oncology study protocols available in the literature, contacts with the study team for technical support (telephonic or via email) are not often considered endpoints. However, these are particularly interesting for feasibility studies, since they provide indirect but real insight on the usability of the platform. Similar to previously published protocols (29–34), our three studies did not include this endpoint, but we believe this omission overlooks valuable data, such as the frequency of unplanned contacts, their reasons, and their resolutions. For instance, if a participant requires (multiple) home visits due to unresolved issues despite telephonic support, this should be taken into consideration in the feasibility analysis, since this information could have a significant impact for healthcare systems or research organizations.

#### 3.1.4 Frequency of active measurements

The question as to how frequent active measurements (such as questionnaires) are acceptable, varies per patient, ranging from once a week to multiple times a day (6). This poses a challenge for research teams when designing studies that balance the need for robust data collection with participant acceptability. This consideration is especially important for studies with long periods of monitoring (e.g., LAPSTAR, 18 months). Though it would be ideal to include a weekly questionnaire for assessing quality of life (QoL) [EORTC QLQ-C30 asks symptoms from last week (35)], repetition over long periods of time could be perceived as burdensome. For the three studies discussed here, we have prioritized minimizing participant burden (thus, for studies such as LAPSTAR with less frequent QoL questionnaires). Interestingly, some participants have proactively contacted the investigator to provide additional information that, they felt, could not be captured by the frequency of the standardized questionnaires. This suggests that additionally to the fixed time points, offering a flexible, non-time-bound option for electronic patient-reported outcomes could support participants who are willing to contribute more detailed information, enhancing the quality of the data without imposing unnecessary burden.

#### 3.1.5 ePRO window

Most electronic and paper patient-reported outcomes follow a predefined assessment schedule. Protocols like eBladder, CHOPIN, and LAPSTAR have varying assessment frequencies, from weekly to specific timepoints. Electronic PROs (ePROs) offer a unique advantage over traditional approaches: the ability to set precise response windows, minimizing data collection deviations (e.g., paper QoL questionnaire planned for week 2 but answered by the participant on week 4). The critical question is determining appropriate ePRO response windows. From our experience, oncological patients often face treatment-related adverse events that may prevent timely questionnaire completion, with a one-day window resulting in considerable missing data. Therefore, we recommend a 3–7-day window, though feasibility of this recommendation varies per study design: while this works for studies like LAPSTAR and CHOPIN (questionnaires every few weeks), weekly questionnaires in eBladder require tighter windows (maximum 3–4 days), otherwise they will overlap with the next questionnaire. This raises important considerations: Should assessment windows be standardized or tailored to each study protocol and patient population? How often should reminder notifications be delivered within a window (e.g., the Promasys ePRO app sends only a single notification at questionnaire release)? Currently, limited literature exists, and therefore, most studies adopt a protocol-specific approach. However, this variability means studies with different response window lengths, for example from 1 to 7 days, may not be directly compared. We believe that standardized mHealth guidelines should exist to ensure consistent data collection and comparability across studies, especially for standardized and validated questionnaires.

### 3.2 Challenges during technology set-up and study execution

#### 3.2.1 Combining treatment and mHealth schedules

One of the most common challenges researchers face when it comes to mHealth studies in oncology is the planning of the study appointments. Synchronizing mHealth study timelines with treatment schedules remains challenging. For instance, a study protocol may specify digital platform installation two weeks before baseline (Figure 2), which seems feasible. However, clinical realities often intervene: systemic therapy or surgery planning can extend over weeks, with final dates frequently set at short notice. This compressed timeline can prevent researchers from completing full baseline assessments, potentially leading to protocol deviations. To address this issue, we recommend initiating data collection at the time of patient consent (Figure 2B), regardless of whether the treatment start date has been determined. Although this approach may lead to a longer baseline data collection period (e.g., 4 weeks instead of 2), it is preferable to the risk of an incomplete baseline (e.g., 1 week or less). To ensure compliance and avoid protocol deviations, this flexibility should be explicitly incorporated into the study protocol.

#### 3.2.2 Unexpected treatment switch and treatment heterogeneity

In oncology, unexpected changes in treatment schemes (e.g., local to systemic) or treatment goals (e.g., curative to palliative) are common. This makes cohort planning complex to follow in practice. The eBladder study exemplifies this complexity: some patients initially stratified in the surgical group (i.e., surgery only), end up receiving adjuvant systemic therapy. Such variations raise critical questions about comparability of recovery trends across different treatment subgroups: are the recovery trends of patients undergoing only radical cystectomy comparable to those getting neo-adjuvant therapy, or those getting systemic treatment (adjuvant) after radical cystectomy comparable to those who got only radio-chemotherapy? These treatment switches are not unique to mHealth studies, they are also present in traditional clinical trials. To address these issues, approaches such as protocol-defined populations, intention-to-treat (ITT), or modified ITT analyses are commonly used in randomized clinical trials to maintain comparability across subgroups (36). In observational studies without digital measurements, treatment changes are addressed using time-varying exposure models, which allow treatment status to change over the course of follow-up and adjust for confounding at each time point (37). These strategies help maintain valid comparisons despite treatment heterogeneity. In mHealth studies, these potential complexities should be carefully considered during study preparation and analysis plan. Consulting an expert panel can also help define precise target cohorts and establish clear inclusion and exclusion criteria. Similarly, developing research data capture tools that are sophisticated enough to account for these treatment variations could be a valuable solution for the future.

#### 3.2.3 Set-up of devices

Regarding device set-up, technical assistance can ease the process for patients. However, cancer patients already endure numerous hospital visits and taxing treatments, so additional appointments should be minimized and ideally combined with existing hospital visits. Home installation by the researcher is an alternative option but increases personnel costs in case of large sample sizes. Postal delivery becomes viable when patients live far from healthcare institutions, lack near-term hospital appointments, and feel technologically confident. In such cases, providing clear set-up instructions and accessible study-team support are critical. Our experience highlights the importance of patient motivation, as some patients receive the devices without ever completing set-up (6). The set-up strategy must balance patient convenience, technological support, and practical constraints of the study protocol; thus, we believe all options should be given in the study protocol to comply with the case-specific factors.

#### 3.2.4 Periodic data checks

A key distinction between traditional clinical trials and at-home mHealth studies lies in the level of participant autonomy. In traditional trials, researchers are primarily responsible for data collection, whereas in mHealth studies, the responsibility shifts almost entirely to the patient. This shift offers advantages, such as reducing recall bias and increasing the objectivity of data. However, it also limits the researchers' control over data quality and consistency. Issues may arise without immediate detection: technology might malfunction unnoticed, participants may become less diligent in recording data, or they may discontinue participation without informing the study team. To mitigate these risks and minimize data loss, we strongly recommend incorporating regular data checks into the assessment schedule (e.g., every three weeks).

#### 3.2.5 End-of-study visit

For the end-of-study (EOS) visit, the ideal approach is that a researcher personally checks and ensures complete data synchronization. When possible, we combine this with existing hospital appointments to minimize patient burden. However, challenges arise when patients live far away or are too frail to travel or receive visits, as often occurs in studies like LAPSTAR. In such cases, returning devices by mail becomes a practical alternative, though it risks data loss: synchronization of the collected data must be checked before deinstallation of devices. Clear instructions and family assistance (in case the patient does not feel confident) are crucial to prevent such data transfer issues. While not optimal, this method provides a necessary flexibility for patient-centric research (2).

### 3.3 Adherence challenges

#### 3.3.1 Integrated platform

Compliance remains a critical challenge in digital remote monitoring studies (38). Developing digital biomarkers and comparing treatment recovery patterns requires large, complete datasets. However, the risk of missing data increases when patient engagement is low—for example, when participants do not take required measurements (such as weight or temperature), do not wear the smartwatch, or do not open the app to allow data to upload. This underscores the importance of aligning study procedures with patient preferences. Patients tend to favor simplified approaches: optimizing apps, for example combining ePRO, appointment tracking, medication reminders, and sensor metrics into a single, tailored application, could significantly improve patient participation and data collection (39). Future research should focus on integrated platforms with minimal hardware and consolidated software solutions (40).

#### 3.3.2 Passive and active measurements

The question of whether passive vs. active measurements impact compliance is complex and individualized. Literature suggests varying results, with some studies indicating that more passive approaches may improve compliance over time (6, 41). In our research, we observe a wide range of patient behaviors: some complete only the minimum required tasks, others less, and some go above and beyond expectations (e.g., in the eBladder and CHOPIN studies, where weekly weight measurements were requested, some patients record them daily). When cancer patients were asked about their preferences (6), most did not have a strong inclination, but generally indicated that a more passive approach might be preferable for longer durations. We believe this issue may be solved by advances in sensor technology, which will provide collection of more endpoints with less active participant effort.

#### 3.3.3 Treatment goals as compliance factor

In oncology, treatment goals (curative vs. palliative) may impact mHealth compliance, and prognosis itself may be even more impactful. For instance, among the palliative groups, 67% (19/28) of CHOPIN participants and 55% (18/33) of LAPSTAR study participants opted for the optional digital monitoring. After randomization, 7 of the 18 participants on the LAPSTAR study were randomized to the control arm, of which 2 (29%) never installed the devices or withdrew within a month. Notably, we have not observed this dropout effect in the intervention arm. This could suggest that disappointment or demoralization associated with being assigned to the control arm in these unblinded studies may reduce motivation to engage with mHealth measurements; an important consideration for researchers designing future randomized mHealth trials.

#### 3.3.4 Assessing mHealth literacy

None of the three studies had an exclusion criterion based on mHealth literacy, and we believe this was the right decision to truly assess the feasibility of such platforms on a population representative of the general oncological public, so including older participants or those less comfortable with technology. However, we did not screen mHealth literacy at the start of the studies, which could have been valuable for analyzing its potential correlation with compliance rates or other outcomes, such as adverse events or healthcare team interactions. Similarly, evaluating socioeconomic status could help determine whether it influences engagement with mHealth technology, an area in which the literature has so far reported inconsistent results (42). Therefore, we recommend assessing both mHealth literacy and socioeconomic status at the start of all mHealth studies. Though there are many ways to assess mHealth literacy, ranging from self-report questionnaires to other structured assessment tools, there is yet no consensus on a universal tool to assess mHealth literacy (43, 44). This remains a significant challenge and an important research gap.

### 3.4 Data reliability

#### 3.4.1 Recording of AEs in real time

As outlined in the protocols of the three studies, adverse events (AEs) were recorded either through direct participant reporting or retrospectively from hospital medical records. However, retrospective data capture may lack objectivity or precision. For example, a participant might report during a follow-up visit that they experienced nausea “last week”, without specifying the exact timing. Accurate temporal information is critical when evaluating the potential of digital technologies to detect AEs and to develop digital biomarkers. To optimize data quality and support biomarker discovery, we advocate for the systemic implementation of patient-reported, real-time AE tracking in future studies.

However, this type of reporting may lead to legal and operational concerns. If providers request such information, they can be held liable for not responding appropriately, or for not creating a continuously available response team (45). In light of these issues, it is important to distinguish between contexts: in clinical trials, real-time AE assessment might be essential for identifying associations with mHealth endpoints and advancing biomarker discovery, whereas in standard clinical care, traditional models, where patients report symptoms and seek help as needed, may remain more practical and sufficient.

#### 3.4.2 Device selection and accuracy

A wide range of mHealth technologies are currently available on the market, which differ in terms of measurement accuracy, available features, pricing, validation status, and user experience (46, 47). Such heterogeneity presents a challenge for researchers when selecting the most appropriate device for a given study. In our view, three key criteria should guide the selection of an mHealth device: [1] Measurement validity and accuracy: devices should be validated against clinical gold standards. Some systematic reviews and validation studies have assessed the reliability and accuracy of various mHealth devices which aids the decision making (48–50). [2] Usability and participant acceptance: user experience and preferences are possibly an important determinant of compliance and long-term adherence; these should be researched preferably before the start of the study through interviews, questionnaires or focus groups (6). [3] Data accessibility and infrastructure compatibility: the devices must be compatible with secure study data storage (e.g., cloud-based systems with audit trails and encryption), allow researchers to access data for data checks, and comply with data protection regulations like the European Union's General Data Protection Regulation (51) or the United States' Health Insurance Portability and Accountability Act (52).

It is important to acknowledge that, while mHealth devices offer opportunities for continuous, real-world monitoring, their accuracy remains generally inferior to that of hospital-grade equipment. For example, consumer sleep trackers often show significant variability compared to polysomnography, especially in detecting sleep stages and wake after sleep onset (53). Nonetheless, mHealth data can provide valuable insights, particularly in long term studies where hospital assessments are impractical or when real-world data is prioritized over clinical precision. Furthermore, the necessity of strict clinical-grade validation can be context-dependent: if mHealth data, despite being maybe less precise in absolute terms, is shown to be reliably associated with relevant health outcomes, and predictive algorithms are explicitly trained to account for device-specific measurement characteristics, then high-fidelity clinical validation may be less critical for certain use cases.

#### 3.4.3 Privacy considerations

The extensive data processing (i.e., collecting, storing, linking) and exchange involved in mHealth, threatens patient's right to private life as enshrined in article 8 of the European Convention of Human Rights (54) and further regulated in the General Data Protection Regulation (51) and national legislation on medical confidentiality. Privacy protection is therefore an important point to be carefully considered in advance. In addition to obtaining participants' consent for data processing in mHealth clinical studies, one of the first measures to be taken in such studies, is pseudonymization. However, this can pose practical challenges. Although researchers can take steps to protect participant identities by assigning pseudonymization codes, there remains a risk of unintentional re-identification. In our studies, for instance, some participants uploaded personal details such as their full name, date of birth, or profile photos to their mHealth study accounts in the consumer-app. This user-generated content can potentially enable third parties to trace data back to individuals, despite the pseudonymization efforts taken by the research staff. To minimize this risk, participants should be clearly instructed at enrollment not to upload any identifiable personal information to the app in situations where this cannot be fully prevented by design. Ideally, clinical apps should be configured with locked-down features that technically restrict such uploads.

See Table 3 for an overview of the discussed challenges in oncology mHealth studies.

**Table 3:** Overview of the challenges of mHealth trials in oncology.

Trial domain	Key challenges
Study set-up and design	Determining appropriate follow-up periods In- and exclusion criteria: gait impairment Contact for digital support as study endpoint Frequency of active measurements Digital questionnaire response windows
Technology set-up and study execution	Combining treatment and mHealth schedules Unexpected treatment switch and treatment heterogeneity Set-up of devices Periodic data checks End-of-study visit
Compliance	Integrated platform Passive and active measurements Treatment goals as compliance factor Assessing mHealth literacy
Data reliability	Recording of AEs in real time Device selection and accuracy Privacy considerations

## 4 Concluding remarks

In this article, we discussed operational challenges encountered in the daily conduct of three mHealth studies in oncology. To our knowledge, this is the first article to discuss such challenges in the oncology field.

We classified these challenges in those encountered during study set-up and design, technology set-up and study execution, adherence challenges, and challenges regarding data reliability, which are summarized in Table 3. We have also provided recommendations as to how some of these challenges can be countered or more efficiently managed.

Strengths of this article include the knowledge of experts in the mHealth field in oncology, and the use of real-world experience from various trials. Limitations include the use of the same platform on the three studies, which may overlook challenges from other platforms. It should also be noted that other challenges undoubtedly exist, but we have only mentioned the most important ones, according to our experience. Similarly, in this article we focused on operational challenges and did not address other relevant aspects, such as the perspectives of healthcare professionals who would ultimately need to interact with these technologies and the resulting data. Although we consider healthcare professionals to be key stakeholders, we intentionally did not include this theme because, in our trials, their involvement was largely limited to the recruitment phase; they did not review the data or discuss it with patients. As a result, we encountered no operational issues related to their role. However, this does not discard the possibility that such challenges may arise once mHealth solutions are fully integrated into standard healthcare practice.

It is important to emphasize that remote monitoring was optional in two of the three studies, which could have influenced engagement in either direction (42). On one hand, patient engagement may have decreased if participants felt that their data were not being regularly reviewed by the clinical team, as this perception can affect motivation (55). On the other hand, those who chose to participate in the optional monitoring component may have been inherently more motivated, potentially resulting in higher engagement.

We believe that further research on mHealth is essential, and that continuous dialogue on emerging challenges, through commentaries, forums, and the sharing of experiences at conferences, is crucial to avoid encountering the same obstacles. Despite this, the potential of mHealth to improve quality of life and perhaps survival outcomes warrant continuous investment and innovation to overcome those challenges. In this article, we highlighted the key operational barriers encountered during the conduct of mHealth studies and propose practical strategies to overcome them. These insights should serve as critical considerations in the rigorous design of future mHealth studies in oncology.

## Data Availability

The original contributions presented in the study are included in the article, further inquiries can be directed to the corresponding author.
